# Kidney function measures and cardiovascular outcomes in people with diabetes: the Hoorn Diabetes Care System cohort

**DOI:** 10.1007/s00125-022-05826-y

**Published:** 2022-11-08

**Authors:** Elisa Dal Canto, Petra J. M. Elders, Amber A. van der Heijden, Adriana J. van Ballegooijen, Birgit I. Lissenberg-Witte, Femke Rutters, Joline W. J. Beulens

**Affiliations:** 1 Department of Experimental Cardiology, University Medical Center Utrecht, Utrecht, the Netherlands; 2 Department of General Practice and Elderly Care Medicine, Amsterdam University Medical Center, Amsterdam, the Netherlands; 3 Department of Epidemiology and Data Science, Amsterdam University Medical Center, Amsterdam, the Netherlands; 4 Julius Center for Health Sciences and Primary Care University Medical Center Utrecht, Utrecht, the Netherlands

**Keywords:** Cardiovascular disease, Diabetes, Kidney function, Time-dependent Cox regression

## Abstract

**Aims/hypothesis:**

Both manifestations of kidney disease in diabetes, reduced eGFR (ml/min per 1.73 m^2^) and increased urinary albumin/creatinine ratio (UACR, mg/mmol), may increase the risk of specific CVD subtypes in adults with diabetes.

**Methods:**

We assessed the prospective association between annually recorded measures of eGFR and UACR and the occurrence of myocardial infarction (MI), CHD, stroke, heart failure (HF) and cardiovascular mortality in 13,657 individuals with diabetes (53.6% male, age 62.3±12.1 years) from the Hoorn Diabetes Care System cohort, using data obtained between 1998 and 2018. Multivariate time-dependent Cox regression models adjusted for cardiovascular risk factors were used to estimate HRs and 95% CI. Associations of eGFR were adjusted for UACR values and vice versa. Effect modification by sex was investigated for all associations.

**Results:**

After a mean follow-up period of 7 years, event rates per 1000 person-years were 3.08 for MI, 3.72 for CHD, 1.12 for HF, 0.84 for stroke and 6.25 for cardiovascular mortality. Mildly reduced eGFR (60–90 ml/min per 1.73 m^2^) and moderately to severely reduced eGFR (<59 ml/min per 1.73 m^2^) were associated with higher risks of MI (HR 1.52; 95% CI 1.10, 2.12 and HR 1.69; 95% CI 1.09, 2.64) and CHD (HR 1.67; 95% CI 1.23, 2.26 and HR 2.01; 95% CI 1.34, 3.02) compared with normal eGFR (>90 ml/min per 1.73 m^2^). Mildly reduced eGFR was associated with a higher risk of stroke (HR 2.53; 95% CI 1.27, 5.03). Moderately increased UACR (3–30 mg/mmol) and severely increased UACR (>30 mg/mmol) were prospectively associated with a higher cardiovascular mortality risk in men and women (HR 1.87; 95% CI 1.41, 2.47 and HR 2.78; 95% CI 1.78, 4.34) compared with normal UACR (<3 mg/mmol). Significant effect modification by sex was observed for the association between UACR and HF. Because there were a limited number of HF events within the category of UACR >30 mg/mmol, categories were combined into UACR <3.0 and >3.0 mg/mmol in the stratified analysis. Women but not men with UACR >3.0 mg/mmol had a significantly higher risk of HF compared with normal UACR (HR 2.79; 95% CI 1.47, 5.28).

**Conclusions/interpretation:**

This study showed differential and independent prospective associations between manifestations of early kidney damage in diabetes and several CVD subtypes, suggesting that regular monitoring of both kidney function measures may help to identify individuals at higher risk of specific cardiovascular events.

**Graphical abstract:**

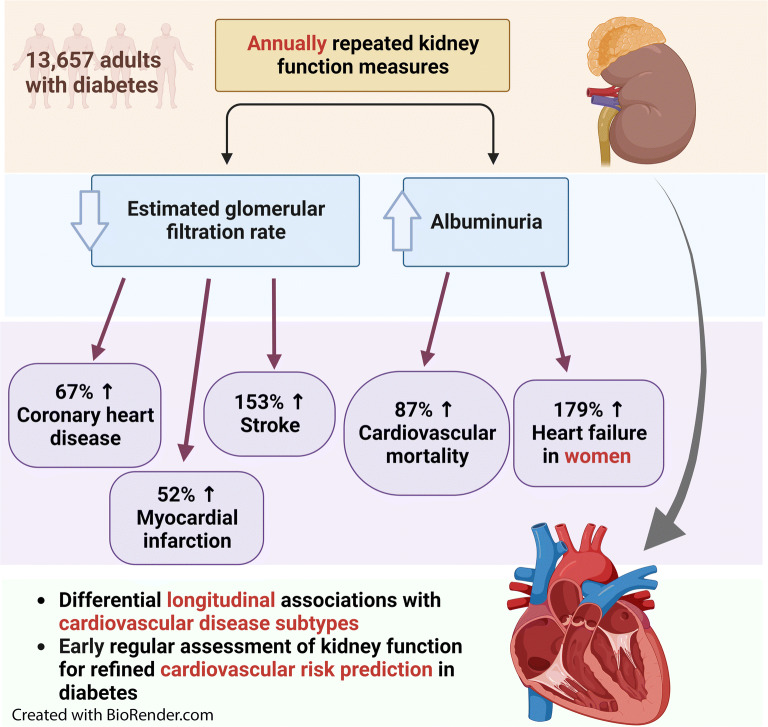

**Supplementary Information:**

The online version of this article 10.1007/s00125-022-05826-y contains peer-reviewed but unedited supplementary material.

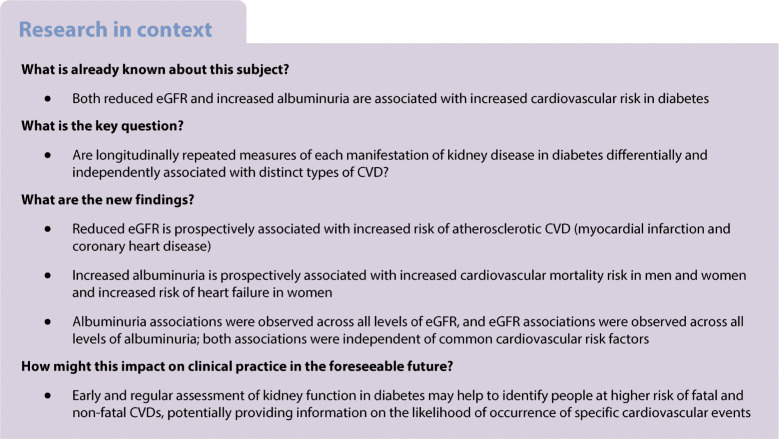



## Introduction

CVD remains the main cause of morbidity and mortality in people with diabetes [1]. However, the epidemiology of CVD in diabetes is changing, with a lower incidence of myocardial infarction (MI) and stroke and higher rates of heart failure (HF) [2, 3]. Cardiovascular risk in diabetes depends on glycaemic control, socioeconomic status and traditional cardiovascular risk factors such as hypertension and dyslipidaemia [4, 5], and further increases with a decline in kidney function [6]. Chronic kidney disease (CKD) develops in approximately 40% of people with diabetes, and adds substantially to their increased mortality risk, which is at least fivefold higher than those with diabetes and preserved kidney function [7].

The two measures of kidney function used to define and stage CKD, the eGFR and urinary albumin/creatinine ratio (UACR), are consistently associated with cardiovascular events and mortality in diabetes [8–15]. However, most studies investigated the association of kidney dysfunction with composite cardiovascular outcomes, rather than distinct CVD subtypes [11, 13, 15]. Moreover, studies mostly focused on atherosclerotic CVD rather than HF, and were performed in relatively small populations [10, 12, 14]. Additionally, in most cases, a single baseline kidney function measure was used, whereas changes in kidney function over time may provide additional information on CVD risk [16, 17]. Albuminuria and reduced eGFR have been shown to be differentially associated with distinct cardiac phenotypes in people with HF with preserved ejection fraction (HFpEF), with and without diabetes [18]. This suggests that eGFR and albuminuria each play a distinct role in cardiovascular health, and increase the risk of specific CVD outcomes through different pathophysiological mechanisms.

Whether early and repeated assessment of kidney function in people with diabetes may help CVD prediction and thus benefit prevention and management of several forms of CVD has not yet been investigated. Accordingly, this study aimed to assess the prospective association of annually repeated measures of eGFR and UACR, the change in each measure over time, and the combination of both, and the occurrence of distinct types of CVD, including MI, CHD, stroke, HF and cardiovascular mortality in people with diabetes.

## Methods

### Study population

**Fig. 1:**
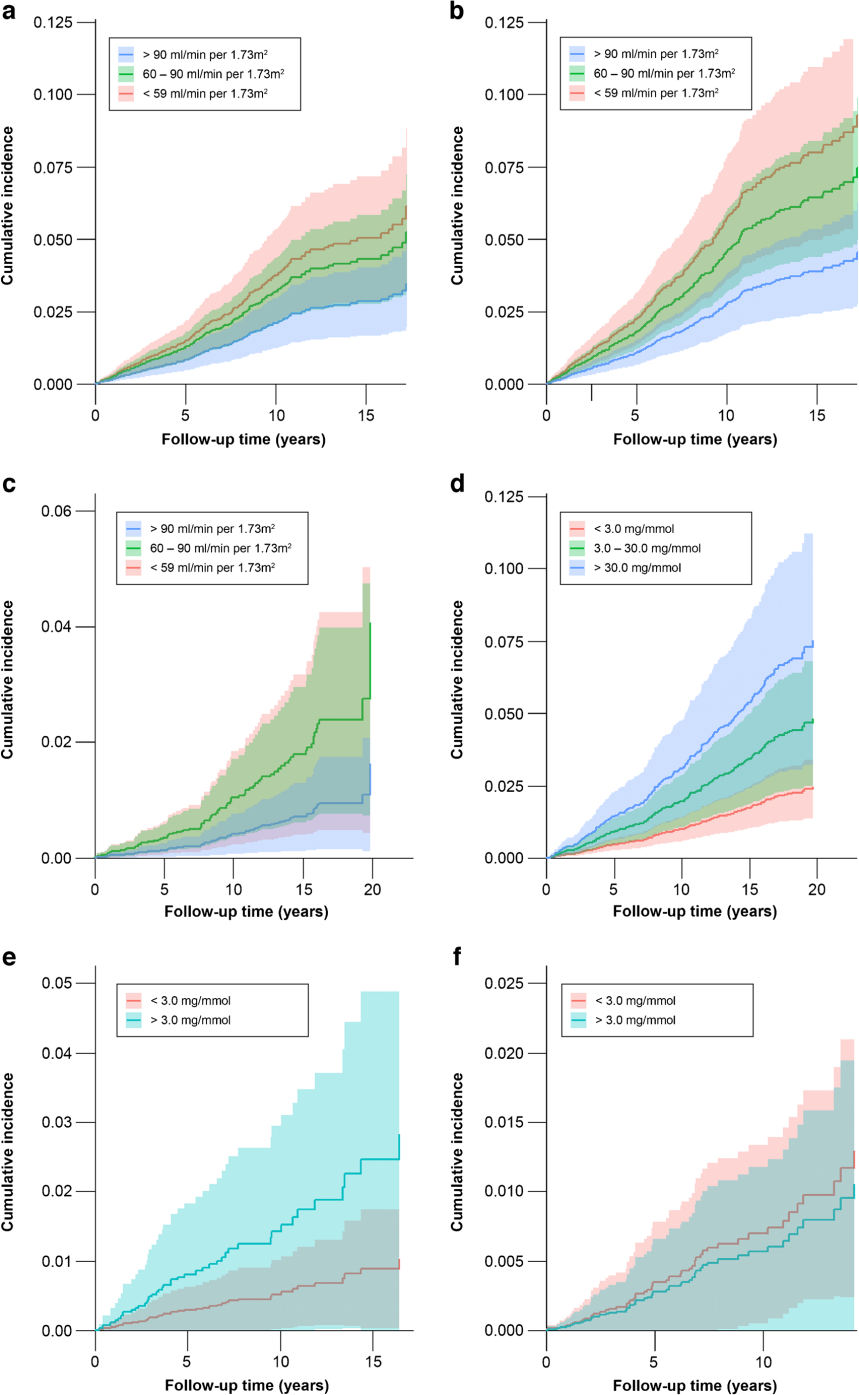
Adjusted cumulative incidence plots of (**a**) MI by eGFR clinical categories, (**b**) CHD by eGFR categories and (**c**) stroke by eGFR categories (only two lines are clearly visible as the categories of eGFR < 59 and 60–90 ml/min per 1.73 m^2^ overlap). (**d**) Cardiovascular mortality by albuminuria categories. (**e**) HF by albuminuria categories in women. (**f**) HF by albuminuria categories in men. All plots are adjusted for model 2 (age, sex, SBP, LDL-cholesterol, HbA_1c_, BMI, duration of diabetes, smoking status and education, use of lipid-lowering therapy, use of ACE inhibitors or angiotensin receptor blockers, any treatment for BP, use of insulin and use of oral hypoglycaemic agents)

This study was performed using data from the Hoorn Diabetes Care System (DCS) cohort, obtained between 1998 and 2018, and described in detail elsewhere [19]. The DCS cohort consists of over 14,000 participants of European ancestry who have diabetes and who visit the centre annually for diabetes monitoring [19]. For the current study, we excluded those without any recorded eGFR values (*n*=129), UACR (*n*=594) or both (*n*=116), resulting in 13,657 participants. In addition, only participants with two or more annual assessments were included. The last observation carried forward method [20] was used to impute missing data for UACR and eGFR (4.1% and 1.5% of annual assessments were missing, respectively). For each cardiovascular outcome, people who had had an event of the same subtype prior to baseline (based on self-reported information) were excluded from the analysis, resulting in different subsamples for each outcome (see electronic supplementary material [ESM] Fig. 1). Diabetes status was confirmed if fasting glucose was ≥7.0 mmol/l on two separate days or ≥7.0 mmol/l with hyperglycaemic symptoms or random glucose was ≥11.0 mmol/l, based on the diagnostic guidelines from the Dutch General Practitioners Association [21]. Type 1 diabetes was defined as onset of diabetes at <40 years and treated with insulin within 4 weeks after the diagnosis. All people included in the study were informed of their inclusion, and agreed to the use of these records for research purposes via an opt-out procedure. The study was approved by the Amsterdam University Medical Center ethics committee, and performed in accordance with the ethical standards stated in the Declaration of Helsinki.

### Kidney function measures

Creatinine concentrations were determined enzymatically using a Cobas c501 Analyzer (Roche Diagnostics, Switzerland), and the eGFR (ml/min per 1.73 m^2^) was calculated using the CKD Epidemiology Collaboration equation [22]. UACR (mg/mmol) was measured on an overnight first-voided urine sample. Urinary creatinine was determined enzymatically, and urinary albumin was determined turbidimetrically using a Cobas c501 Analyzer (Roche Diagnostics) [19]. Based on the CKD Epidemiology Collaboration definitions, eGFR stages (ml/min per 1.73 m^2^) were defined as: G1, normal to high (≥ 90); G2, mildly decreased (60–89); G3a, mildly to moderately decreased (45–59); G3b, moderately to severely decreased (30–44); G4, severely decreased (15–29); G5, kidney failure (<15). Information on dialysis status was not recorded, although it is likely that most G5 stage participants (0.1%) were on dialysis. Albuminuria stages were defined as: A1, normal to mildly increased UACR (<3.0 mg/mmol); A2, moderately increased UACR (3.0–30.0 mg/mmol); A3, severely increased UACR (>30.0 mg/mmol) [23].

### Cardiovascular outcomes

Cardiovascular outcomes were defined as the first occurrence of a non-fatal cardiovascular event and were self-reported during the annual visit. Events that occurred before baseline were also self-reported. The reports were verified against electronic medical registrations from regional hospital and general practitioner records in a subsample of 453 participants; this comparison showed a sensitivity and specificity of 86% and 90%, respectively [19]. Cardiovascular events were coded according to the International Classification of Diseases 9th (ICD-9) revision (http://www.icd9data.com/2007/Volume1/default.htm) as MI (ICD 410–411), CHD (410–414), stroke (435) and HF (428–429). Vital status was checked every 6 months using the national population registry, and cause of death was determined using general practitioner records and the morbidity and mortality registry. The cardiovascular mortality outcome was defined as death from any CVD (ICD 390–459).

### Covariates

Fasting plasma glucose, HbA_1c_, plasma total cholesterol, HDL-cholesterol and LDL-cholesterol concentrations were determined as described previously [19]. BMI was calculated by dividing body weight by height squared (kg/m^2^). Systolic BP (SBP) and diastolic BP (DBP) were measured according to a standardised protocol [19], and arterial hypertension was defined as SBP ≥140 mmHg, DBP ≥90 mmHg and/or use of anti-hypertensive medication. Information on current medication use was registered by medication inventory. Information on diabetes duration, education and smoking status was obtained by self-report. High education level was defined as higher vocational education or university, medium education level was defined as secondary education, and low education level was defined as elementary school, lower vocational training or less.

### Statistical analysis

All analyses were performed using SPSS Statistics version 22.0 (IBM, USA) and R-Studio version 3.4.2 (R Foundation for Statistical Computing, Austria). The association between eGFR, UACR and cardiovascular outcomes was investigated using multivariate time-dependent Cox regression. All variables, apart from sex and education, were longitudinally recorded and treated as time-dependent, and an extended Cox model was applied to allow the values to change over time [24]. A minimally adjusted model included age (years), sex, SBP (mmHg), LDL-cholesterol (mmol/l), HbA_1c_ (%), BMI (kg/m^2^), diabetes duration (years), smoking status (never/former/current) and education level (high/low/medium) (model 1). A second model additionally included use of lipid-lowering agents, use of ACE inhibitors or angiotensin receptor blockers, use of any anti-hypertensive medication and use of insulin or oral hypoglycaemic agents (yes/no) (model 2). The fully adjusted model additionally included UACR (mg/mmol) for the analyses between eGFR and CVD outcomes, and eGFR (ml/min per 1.73 m^2^) for the analyses between UACR and CVD outcomes (model 3).

Both eGFR and UACR were analysed as clinical categories and as continuous variables. Clinical categories were based on the CKD Epidemiology Collaboration definitions; however, as stages of more advanced kidney dysfunction (eGFR <45 ml/min per 1.73 m^2^) were scarcely represented in our sample, we used three categories of eGFR: >90, 60–89 and <59 ml/min per 1.73 m^2^. Likewise, we used UACR categories of <3.0, 3.0–30.0 and >30.0 mg/mmol. The categories of eGFR >90 ml/min per 1.73 m^2^ and UACR <3.0 mg/mmol were used as reference categories. UACR and eGFR were combined into risk categories based on the Kidney Disease: Improving Global Outcomes (KDIGO) classification [23]. Because the ‘very high risk’ category was scarcely represented, it was combined with the ‘high risk’ category, resulting in ‘low’, ‘moderate’ and ‘high risk’ categories.

For the continuous analysis of kidney function measures, we created a categorical variable using negative steps of eGFR equal to 10 ml/min per 1.73 m^2^ and log-transformed UACR because of its skewed zero inflated distribution. Trajectories for eGFR and UACR were studied by calculating their longitudinal change over time. The absolute annual change in eGFR and UACR was calculated as the difference between each visit and the previous visit, divided by the time interval between assessments, and was included in the Cox regression model as a time-varying variable. A *p* value <0.05 was considered statistically significant.

Sex was assessed as an effect modifier by including interaction terms with eGFR and UACR, both as continuous variables and as clinical categories, in the fully adjusted models, in addition to the main effects. Likewise, interaction between eGFR and UACR as continuous variables and as categories was assessed. Wald tests were used to assess whether the models with the interaction terms differed from those without. A *p* value <0.10 for the interaction was considered statistically significant.

Event rates were calculated as the number of events divided by the total person-years of follow-up, and expressed as the number per 1000 person-years. Annual assessments of the covariates were missing in proportions ranging from 0.9% for diabetes duration to 10.4% for education, and were imputed using the last observation carried forward method [20]. A complete-case analysis was performed for the association between eGFR and UACR categories and CVD outcomes, and the results were compared with the main analysis. Further, a conventional Cox regression analysis was performed to investigate the prospective association between baseline kidney function measures as categories and CVD outcomes. Sensitivity analyses were conducted by (1) excluding all individuals with any CVD prior to baseline, regardless of the outcome; and (2) excluding those with diabetes types other than type 2.

## Results

### Baseline clinical characteristics

We included 13,657 individuals with a mean age at baseline of 62.3±12.1 years, of whom 98.9% had type 2 diabetes and 53.6% were men. The distribution of eGFR was approximately normal, with a mean value of 80.4±18.8 ml/min per 1.73 m^2^, whereas UACR showed a skewed distribution, with a median value of 0.6 mg/mmol (IQR 0.3–1.4) (ESM Table 1). Most people at baseline had a normal or mildly reduced eGFR and normal to mildly increased UACR (ESM Table 2). The median number of assessments was five (IQR 2–8), and the median annual changes in eGFR and UACR were 0.1 ml/min per 1.73 m^2^ (IQR −2.0 to 0.4) and −1.4 mg/mmol (IQR −5.6 to 2.8), respectively. During follow-up periods that ranged from 4.6 years (IQR 1.7–7.6) for MI to 7.9 years (IQR 3.3–11.4) for stroke, the median changes in eGFR and UACR from baseline were −8.1 ml/min per 1.73 m^2^ (IQR −17.1 to −1.1) and 0.3 mg/mmol (IQR −0.1 to 1.1), respectively.

Compared to people with normal eGFR, those with mildly reduced and moderately to severely reduced eGFR were older, less likely to be male, more often hypertensive, less often obese, and used cardiovascular drugs more frequently (Table 1). Stratifying people by albuminuria categories yielded a similar gradient in terms of older age, comorbidities and use of cardiovascular drugs. However, those with increased UACR were more likely to be male and obese compared to those with normal UACR (ESM Table 3). The overall proportion of people requiring anti-hyperglycaemic medications did not clearly change across eGFR categories. However, the utilisation of oral anti-hyperglycaemic agents, and particularly of metformin, decreased with reduced eGFR, while insulin use increased for moderately to severely reduced eGFR (Table 1). For UACR categories, the proportion of total anti-hyperglycaemic medications, and particularly insulin use, increased with increased albuminuria (ESM Table 3).

**Table 1:** Baseline characteristics of 13,657 individuals with diabetes stratified by eGFR categories

	eGFR category (min/ml per 1.73 m^2)^		
	≥90	60–90	<59
*n*	4396	7376	1885
Age (years)	53.0 ± 10.3	65.0 ± 10.0	73.3 ± 8.5
Male (%)	58.9	53.5	41.6
Smoking status			
Current smokers (%)	31.1	16.8	12.4
Former smokers (%)	33.6	40.8	34.7
Education level			
Low	36.9	44.4	55.4
Medium	44.7	39.6	33.3
High	17.6	15.7	10.4
Type 2 diabetes (%)	97.9	99.4	99.6
Type 1 diabetes (%)	1.6	0.4	0.2
Other diabetes types (MODY, LADA, etc)	0.5	0.2	0.2
Diabetes duration (years)	0.5 (0.1–2.5)	0.7 (0.1–3.4)	1.1 (0.2–5.4)
HbA_1c_ (mmol/mol)	58 ± 9	54 ± 7	54 ± 7
HbA_1c_ (%)	7.5 ± 1.7	7.1 ± 1.4	7.1 ± 1.4
Comorbidities			
UACR (mg/mmol)	0.5 (0.3–1.3)	0.5 (0.3–1.2)	0.9 (0.4–3.5)
Arterial hypertension (%)	44.2	58.6	65.4
Brachial BP (mmHg)			
Systolic	137 ± 19	144 ± 21	149 ± 24
Diastolic	81 ± 10	81 ± 10	79 ± 11
Obesity (%)	49.4	42.3	44.3
BMI (kg/m^2^)	30.7 ± 6.0	29.9 ± 5.2	30.1 ± 5.1
Total serum cholesterol (mmol/l)	5.0 ± 1.1	5.1 ± 1.2	5.0 ± 1.2
Serum HDL-cholesterol (mmol/l)	1.2 ± 0.3	1.2 ± 0.4	1.2 ± 0.3
Serum LDL-cholesterol (mmol/l)	2.9 ± 1.0	3.0 ± 1.0	2.9 ± 1.1
Medications			
Anti-hypertensive medications			
ACE inhibitors/ARBs (%)	25.5	32.5	49.0
Beta-blockers (%)	22.1	31.9	46.9
Calcium antagonists (%)	9.6	14.8	22.0
Diuretics (%)	22.4	22.7	44.2
Lipid-lowering medications (%)	35.4	40.9	47.2
Anti-hyperglycaemic medications (%)	73.5	66.7	69.1
Oral anti-hyperglycaemic medications (%)	61.6	58.4	55.1
Metformin	59.5	50.1	45.5
Sulfonylureas	22.2	23.5	26.8
Dipeptidyl peptidase-4 inhibitors	0.5	0.4	0.6
Glucagon-like peptide 1 receptor agonists	0.3	0.05	0.0
Sodium–glucose cotransporter 2 inhibitors	0.1	0.1	0.0
Thiazolidinediones	0.2	0.3	0.3
Insulin use (%)	12.0	8.3	13.9

Values are means ± SD, percentages or median (IQR)

ARBs, angiotensin receptor blockers; LADA, latent autoimmune diabetes in adults

### Associations between longitudinal measures of kidney function and cardiovascular outcomes

Event rates per 1000 person-years were 3.08 for MI, 3.72 for CHD, 0.84 for stroke and 1.12 for HF (Table 2). The risk of MI was significantly higher for mildly reduced eGFR (HR 1.52; 95% CI 1.10, 2.12) and for moderately to severely reduced eGFR (HR 1.69; 95% CI 1.09, 2.64) compared with normal eGFR, independently of cardiovascular risk factors, medication use and UACR values. Similarly, mildly reduced and moderately to severely reduced eGFR showed higher risks of CHD (HR 1.67; 95% CI 1.23, 2.26 and HR 2.01; 95% CI 1.34, 3.02, respectively), whereas only mildly reduced eGFR was significantly associated with higher risk of stroke (HR 2.53; 95% CI 1.27, 5.03) (model 3; Table 2 and Fig. 1a–c). For albuminuria, only severely increased UACR was associated with a higher risk of MI, independently of cardiovascular risk factors, medication use and eGFR values; no significant associations were observed between albuminuria and HF, CHD or stroke (Table 2, model 3).

**Table 2:** HRs of MI, HF, stroke, CHD and cardiovascular mortality in individuals with diabetes stratified by eGFR and albuminuria clinical categories

	MI			HF			Stroke			CHD			Cardiovascular mortality		
Number of events	325			127			95			408			686		
Event rate per 1000 person-years^a^	3.08			1.12			0.84			3.72			6.25		
	HR	95% CI	*p* value	HR	95% CI	*p* value	HR	95% CI	*p* value	HR	95% CI	*p* value	HR	95% CI	*p* value
Model 1															
eGFR > 90 ml/min per 1.73 m^2^	Reference			Reference			Reference			Reference			Reference		
eGFR 60–90 ml/min per 1.73 m^2^	1.54	1.11, 2.15	0.010	1.39	0.76, 2.57	0.284	2.44	1.23, 4.84	0.010	1.69	1.25, 2.30	<0.001	0.81	0.50, 1.32	0.407
eGFR < 59 ml/min per 1.73 m^2^	1.78	1.15, 2.76	0.009	1.84	0.88, 3.83	0.103	2.32	0.94, 5.68	0.066	2.04	1.37, 3.04	<0.001	1.72	1.02, 2.90	0.040
UACR < 3.0 mg/mmol	Reference			Reference			Reference			Reference			Reference		
UACR 3.0–30.0 mg/mmol	1.05	0.75, 1.47	0.772	1.30	0.77, 2.20	0.321	1.30	0.76, 2.24	0.341	0.99	0.73, 1.35	0.970	2.14	1.62, 2.83	<0.001
UACR > 30.0 mg/mmol	2.09	1.25, 3.48	0.005	1.97	0.70, 4.93	0.144	1.78	0.72, 4.41	0.213	1.70	1.04, 2.78	0.034	3.56	2.28, 5.55	<0.001
Model 2															
eGFR > 90 ml/min per 1.73 m^2^	Reference			Reference			Reference			Reference			Reference		
eGFR 60–90 ml/min per 1.73 m^2^	1.54	1.11, 2.14	0.010	1.40	0.76, 2.57	0.275	2.55	1.28, 5.06	0.008	1.68	1.24, 2.28	<0.001	0.74	0.46, 1.20	0.223
eGFR < 59 ml/min per 1.73 m^2^	1.80	1.15, 2.81	0.010	1.81	0.87, 3.73	0.108	2.50	0.99, 6.28	0.051	2.09	1.40, 3.12	<0.001	1.40	0.84, 2.35	0.194
UACR < 3.0 mg/mmol	Reference			Reference			Reference			Reference			Reference		
UACR 3.0–30.0 mg/mmol	1.04	0.74, 1.45	0.836	1.27	0.77, 2.14	0.367	1.33	0.76, 2.34	0.317	0.99	0.73, 1.35	0.954	1.95	1.47, 2.57	<0.001
UACR > 30.0 mg/mmol	2.08	1.25, 3.45	0.004	1.93	0.877, 4.84	0.159	1.94	0.72, 4.87	0.159	1.70	1.04, 2.78	0.033	3.16	2.02, 4.85	<0.001
Model 3															
eGFR > 90 ml/min per 1.73 m^2^	Reference			Reference			Reference			Reference			Reference		
eGFR 60–90 ml/min per 1.73 m^2^	1.52	1.10, 2.12	0.022	1.40	0.77, 2.57	0.270	2.53	1.27, 5.03	0.008	1.67	1.23, 2.26	0.001	0.75	0.46, 1.20	0.227
eGFR < 59 ml/min per 1.73 m^2^	1.69	1.09, 2.64	0.012	1.73	0.84, 3.56	0.135	2.32	0.91, 5.94	0.078	2.01	1.34, 3.02	<0.001	1.29	0.76, 2.14	0.349
UACR < 3.0 mg/mmol	Reference			Reference			Reference			Reference			Reference		
UACR 3.0–30.0 mg/mmol	1.03	0.73, 1.46	0.850	1.26	0.74, 2.12	0.392	1.35	0.77, 2.35	0.293	0.98	0.72, 1.34	0.907	1.87	1.41, 2.47	<0.001
UACR > 30.0 mg/mmol	1.98	1.18, 3.30	0.009	1.81	0.73, 4.55	0.201	1.87	0.72, 4.83	0.195	1.57	0.95, 2.59	0.077	2.78	1.78, 4.34	<0.001

^a^Event rates were calculated as the number of events divided by the total person-years of follow-up, and expressed as the number per 1000 person-years

Model 1 is adjusted for age, sex, SBP, LDL-cholesterol, HbA_1c_, BMI, duration of diabetes, smoking status and education. Model 2 is additionally adjusted for lipid-lowering therapy, use of ACE inhibitors or angiotensin receptor blockers, any treatment for arterial hypertension, use of insulin and use of oral hypoglycaemic agents. Model 3 is adjusted as for model 2 with the addition of albuminuria categories for the analysis of eGFR or eGFR categories for the analysis of albuminuria

Significant effect modification by sex was observed for the association between albuminuria and HF. Because of a limited number of HF events within the category UACR >30 mg/mmol in both sexes, categories were combined into UACR <3.0 and >3.0 mg/mmol in the stratified analysis (*p*_interaction_ = 0.010). In men, no significant associations were observed between UACR and HF. However, women with UACR >3.0 mg/mmol had a significantly higher risk of HF than those with normal UACR, independently of cardiovascular risk factors, medication use and eGFR values (HR 2.79; 95% CI 1.47, 5.28) (model 3; Table 3 and Fig. 1e,f).

**Table 3:** HRs for HF in men and women with diabetes stratified by albuminuria clinical categories

	Men			Women		
Number of events	69			58		
Event rate per 1000 person-years^a^	1.16			1.06		
	HR	95% CI	*p* value	HR	95% CI	*p* value
Model 1						
UACR < 3.0 mg/mmol	Reference			Reference		
UACR > 3.0 mg/mmol	0.83	0.41, 1.67	0.597	2.74	1.47, 5.14	0.002
Model 2						
UACR < 3.0 mg/mmol	Reference			Reference		
UACR > 3.0 mg/mmol	0.79	0.39, 1.59	0.513	2.71	1.45, 5.07	0.002
Model 3						
UACR < 3.0 mg/mmol						
UACR > 3.0 mg/mmol	0.78	0.39, 1.54	0.474	2.79	1.47, 5.28	0.002

^a^Event rates were calculated as the number of events divided by the total person-years of follow-up, and expressed as the number per 1000 person-years.

Because of the very limited number of HF events within the category UACR > 30.0 mg/mmol in both sexes, the categories UACR = 3.0–30.0 and UACR > 30.0 have been combined into one category for the stratified analysis

Effect modification by sex for the association between HF and albuminuria categories: interaction dummy for UACR category > 3.0 mg/mmol, *p* = 0.010

Model 1 is adjusted for age, sex, SBP, LDL-cholesterol, HbA_1c_, BMI, duration of diabetes, smoking status and education. Model 2 is additionally adjusted for lipid-lowering therapy, use of ACE inhibitors or angiotensin receptor blockers, any treatment for arterial hypertension, use of insulin and use of oral hypoglycaemic agents. Model 3 is adjusted as for model 2 with the addition of albuminuria categories for the analysis of eGFR or eGFR categories for the analysis of albuminuria

For the analysis of longitudinal change over time, a slower annual decrease in eGFR was significantly associated with reduced HF risk in men and women (HF risk decreased by 17% for every 1 ml/min per 1.73 m^2^ annual increase in eGFR), while a more rapid annual increase in UACR was significantly associated with higher HF risk in men (HF risk in men increased by 14% for every 1 mg/mmol annual increase in UACR) (Tables 4 and 5). When eGFR and UACR were analysed continuously, reduced eGFR was significantly associated with higher risks of CHD and HF, while increased UACR was significantly associated with higher risk of stroke (ESM Tables 4 and 5).

**Table 4:** HRs for MI, HF, stroke and CHD in individuals with diabetes for absolute annual change in eGFR

	Model 1			Model 2			Model 3		
	HR	95% CI	*p* value	HR	95% CI	*p* value	HR	95% CI	*p* value
MI	0.91	0.81, 1.01	0.068	0.90	0.81,1.01	0.073	0.90	0.82, 1.01	0.073
HF	0.83	0.78, 0.87	<0.001	0.82	0.76, 0.87	<0.001	0.82	0.76, 0.87	<0.001
HF, men	0.86	0.81, 0.91	<0.001	0.86	0.80, 0.92	<0.001	0.86	0.80, 0.92	<0.001
HF, women	0.55	0.46, 0.64	<0.001	0.54	0.45, 0.64	<0.001	0.54	0.45, 0.64	<0.001
Stroke	1.12	0.58, 2.16	0.736	1.12	0.59, 2.14	0.729	1.12	0.59, 2.14	0.728
CHD	0.96	0.74, 1.25	0.758	0.96	0.74, 1.25	0.790	0.97	0.74, 1.15	0.790
Cardiovascular mortality	0.80	0.76, 0.84	<0.001	0.80	0.75, 0.84	<0.001	0.79	0.75, 0.85	<0.001

The absolute annual change in eGFR was calculated as the difference between consecutive assessments divided by the time interval between assessments, and was included in the Cox regression model as a time-varying variable

Model 1 is adjusted for age, sex, SBP, LDL-cholesterol, HbA_1c_, BMI, duration of diabetes, smoking status and education. Model 2 is additionally adjusted for lipid-lowering therapy, use of ACE inhibitors or angiotensin receptor blockers, any treatment for arterial hypertension, use of insulin and use of oral hypoglycaemic agents. Model 3 is adjusted as for model 2 plus absolute annual change in UACR

**Table 5:** HRs for MI, HF, stroke and CHD in individuals with diabetes for the absolute annual change^a^ in UACR

	Model 1			Model 2			Model 3		
	HR	95% CI	*p* value	HR	95% CI	*p* value	HR	95% CI	*p* value
MI	1.05	0.95, 1.16	0.334	1.05	0.95, 1.16	0.331	1.05	0.95, 1.16	0.333
HF	1.08	1.05, 1.12	<0.001	1.07	1.03, 1.11	<0.001	1.07	1.04, 1.12	<0.001
HF, men	1.14	1.08, 1.22	<0.001	1.13	1.07, 1.20	<0.001	1.14	1.07, 1.21	<0.001
HF, women	1.05	0.89, 1.24	0.570	1.06	0.89, 1.27	0.502	1.05	0.90, 1.23	0.505
Stroke	0.94	0.91, 0.97	<0.001	0.94	0.91, 0.97	<0.001	1.04	0.95, 1.13	0.434
CHD	1.03	0.97, 1.09	0.377	1.03	0.97, 1.09	0.343	1.04	0.96, 1.15	0.437
Cardiovascular mortality	1.07	1.02, 1.13	0.007	1.07	1.01, 1.31	0.012	1.07	1.01, 1.13	0.002

^a^The absolute annual change in UACR was calculated as the difference between consecutive assessments divided by the time interval between assessments, and was included in the Cox regression model as a time-varying variable

Model 1 is adjusted for age, sex, SBP, LDL-cholesterol, HbA_1c_, BMI, duration of diabetes, smoking status and education. Model 2 is additionally adjusted for lipid-lowering therapy, use of ACE inhibitors or angiotensin receptor blockers, any treatment for arterial hypertension, use of insulin and use of oral hypoglycaemic agents. Model 3 is adjusted as for model 2 plus absolute annual change in eGFR

An interaction was observed between the categories UACR >3 mg/mmol and eGFR <60 ml/min per 1.73 m^2^ and HF risk in men but not women (*p*_interaction_ = 0.072). Interactions between eGFR and UACR as continuous variables were observed for MI (*p*=0.077) and CHD risks (*p*=0.097) in men and women, and with HF only in women (*p*=0.097). However, despite the interactions, there were no significant changes in the main effects.

### Associations between longitudinal measures of kidney function and cardiovascular mortality

After follow-up for 6.4 years (IQR 3.6–10.4) the cardiovascular mortality rate was 6.25 per 1000 person-years. Compared to those with normal eGFR, people with moderately to severely reduced eGFR showed higher cardiovascular mortality risk but the association was no longer significant in the fully adjusted model. On the other hand, both people with moderately increased UACR (HR 1.87; 95% CI 1.41, 2.47) and severely increased UACR (HR 2.78; 95% CI 1.78, 4.34) had a significantly higher cardiovascular mortality risk, independently of cardiovascular risk factors, medication use and eGFR values (model 3; Table 2 and Fig. 1d). Analysis of longitudinal changes indicated that a slower decrease in eGFR was significantly associated with a lower cardiovascular mortality risk, while a more rapid increase in UACR was significantly associated with a higher cardiovascular mortality risk (Tables 4 and 5). Similarly, both reduced eGFR and increased UACR as continuous variables were significantly associated with higher cardiovascular mortality risk (ESM Tables 4 and 5).

### Additional analyses

None of the risk categories of combined eGFR and UACR showed any significant associations with any of the CVD subtypes. Conversely, both moderate and high risk categories showed higher cardiovascular mortality risk compared with the low risk category (ESM Table 6). Cox regression analysis of the association between baseline kidney function categories and CVD events mostly led to smaller effect sizes and non-significant associations (ESM Table 7). Excluding all individuals with any CVD prior to baseline and excluding individuals with types of diabetes other than type 2 diabetes did not materially change our findings (ESM Tables 8 and 9). The complete-case analysis gave results that were comparable to those for the analysis on the imputed dataset (ESM Tables 10 and 11).

## Discussion

In this study, longitudinal measures of eGFR and albuminuria were differentially associated with distinct CVD subtypes and cardiovascular mortality in people with diabetes, independently of cardiovascular risk factors and cardiovascular drug use. Importantly, we found that eGFR and albuminuria associations with CVD subtypes were independent of each other. In particular, individuals with mildly reduced and with moderately to severely reduced eGFR showed higher risks of MI and CHD compared to those with normal eGFR, and those with mildly reduced eGFR showed higher risk of stroke. Women but not men with increased albuminuria had higher HF risk than those with no albuminuria. Finally, individuals with severely increased albuminuria had higher risk of MI and those with moderately and severely increased albuminuria had higher cardiovascular mortality risk than those with no albuminuria.

Analyses of eGFR and UACR as continuous variables and as an annual change yielded different and often weaker associations than those for eGFR and UACR categories. However, the association between either eGFR and UACR and cardiovascular mortality was present consistently. This may be due to the distribution of kidney function in our population, with a small proportion of individuals having advanced CKD, the small annual variation in both kidney function measures of most patients, and the relatively low number of cardiovascular events of each type, with the exception of cardiovascular mortality.

Overall, our findings are consistent with previous studies that established kidney disease as a strong independent risk factor for CVD in diabetes [11, 13, 14, 25, 26]. These studies mostly assessed the association between the baseline presence of CKD and future risk of CVD; however, kidney function may vary over time. Only a few studies investigated the association between the progression of kidney function decline and future risk of CVD in people with diabetes [16, 17]. These studies found that a more rapid increase in UACR and a more rapid decrease in eGFR over time were associated with 3.15 and 4.11-fold higher risks of cardiovascular events and mortality respectively, compared with slower changes [17]. However, the outcome of interest was a composite of major cardiovascular events rather than specific CVD subtypes. To our knowledge, our study is the first to assess the differential associations between longitudinal measures of eGFR and UACR and distinct cardiovascular events in a large cohort of people with diabetes.

Our results indicated differential and independent associations between each manifestation of early kidney disease in diabetes and CVD. The association between reduced eGFR and atherosclerotic CVD and cardiovascular mortality has been repeatedly reported previously [12, 14, 17]. Increased albuminuria has been shown to be associated with higher risks of atherosclerotic CVD, cardiovascular mortality [13, 14], HFpEF and HF with reduced ejection fraction (HFrEF) [25], even in the absence of reduced eGFR [27]. Diabetes contributes to accelerated atherosclerosis, and atherosclerotic lesions start to develop in early stages of CKD [28]. Reduced eGFR leads to endothelial dysfunction, oxidative stress, hypercoagulability and inhibition of erythropoiesis and platelet function, which favour vascular calcification and atherogenesis, thereby explaining its strong link with atherosclerotic CVD [29]. Importantly, reduced eGFR exerts its harmful effect via pathways that are independent of traditional risk factors. On the other hand, albuminuria represents an independent risk marker of diabetic microangiopathy, and increases as a consequence of the activation of the renin–angiotensin–aldosterone system [30]. Hyperactivation of this system raises left ventricular workload and induces coronary endothelial microvascular dysfunction, leading to left ventricular hypertrophy, diastolic dysfunction and HF, particularly HFpEF [29]. However, an association between albuminuria and HF was only present in women. Plausible explanations are the predisposition of elderly women with diabetes to develop HF, and especially HFpEF, more often than men [31], together with the presence of an overly activated renin–angiotensin–aldosterone system in response to low oestrogen levels [31].

Increased UACR, both as categories and as a continuous variable, reduced eGFR as a continuous variable, and moderate and high risk categories of combined eGFR and UACR were independently associated with a higher cardiovascular mortality risk. Further, a more rapid decrease in eGFR and more rapid increase in UACR over time were significantly associated with higher cardiovascular mortality risk. This is in agreement with previous studies, as kidney dysfunction is a well-known pathogenic factor that results in abnormal cardiac mechanics and vascular damage, and consequently may lead to death from CVD [29].

When only the baseline value of kidney function measures was taken into account, and analysed in association with the occurrence of CVD outcomes using a conventional Cox regression method, most associations were not significant. This may potentially be explained by the majority of participants having normal kidney function at baseline. This finding further highlights the importance of a regular assessment of kidney function in people with diabetes. Monitoring the changes in eGFR and albuminuria over time may help to identify participants at higher risk of both fatal and non-fatal CVDs, potentially providing information on the likelihood of occurrence of specific cardiovascular events. Interestingly, our results indicated that UACR associations with HF in women and with cardiovascular mortality in men and women were present across all levels of eGFR. Likewise, eGFR associations with MI and CHD were observed across all levels of UACR. This indicates multiplicative independence, and suggests that eGFR and albuminuria may play distinct roles in cardiovascular pathophysiology, thereby driving the development of different CVD subtypes.

Previous studies indicated that addition of eGFR and UACR to prediction models consisting of traditional risk factors improved cardiovascular risk prediction in people with diabetes, especially for cardiovascular mortality (change in C statistic = 0.0139; 95% CI 0.0105, 0.0174) and HF (change in C statistic = 0.0196; 95% CI 0.0108, 0.0284) [6]. As current cardiovascular risk scores do not perform well in people with diabetes [32], future studies should test and validate the implementation of kidney function measures for refined cardiovascular risk assessment. Individuals at higher cardiovascular risk could then be eligible for prevention strategies, such as treatment with sodium–glucose cotransporter 2 inhibitors.

Some limitations need to be addressed. First, to maximise the number of events, we excluded only those people who did not have an event of the same type prior to baseline for each outcome. Consequently, people with an established CVD of a different kind were still included. This may have biased some of the associations, as those people may have had a higher risk of subsequent events. However, a sensitivity analysis excluding individuals with any kind of prior CVD did not significantly change our findings. Second, diagnoses of CVD were not always mutually exclusive, and in some cases overlapped, as an individual with MI could at the same time also be included in the CHD and/or in the HF category. Further, the self-reporting of cardiovascular events may have been inaccurate in some cases, leading to outcome misclassification. Third, inadequate statistical power may have accounted for the lack of significance of some of the associations. Our findings should therefore be confirmed in a larger population with higher event rates. Further, in some of the analyses, we combined categories of eGFR and UACR, because of a minor proportion of people having advanced CKD. This has prevented assessment of cardiovascular risk in people with severe CKD. However, it is acknowledged that such individuals are at considerably high cardiovascular risk, whereas a strong point of our findings is that even mild impairment of kidney function is associated with a wide range of cardiovascular events. Fourth, lifestyle factors such as alcohol consumption, diet quality and level of physical activity, and information on socioeconomic status other than educational level, are not routinely recorded in the Hoorn DCS. Some residual confounding by such factors may be present and may have affected our findings. Fifth, we could not investigate the association between CKD and the risk of HFrEF and HFpEF separately, as we were unable to ascertain the HF subtype. However, because HF in diabetes currently occurs more frequently as HFpEF than HFrEF, we assume that a reasonable proportion of HF events in this study were actually HFpEF. Future studies should investigate whether a reduced eGFR and especially albuminuria may also predict the risk of developing HFpEF in individuals with diabetes for better targeted prevention/management. Finally, by using time-dependent Cox regression, we allowed for changes in eGFR and UACR over time and accounted for variation in confounders, but could not investigate whether kidney function measurements performed earlier or those performed later during the course of the disease were more useful for cardiovascular risk prediction. Accordingly, we could not address the issue of having different numbers of annual assessments. In this regard, future studies using joint modelling analysis, are needed on this topic.

Strengths of our study include the relatively large sample size, its prospective design with multiple standardised annual measurements for each patient, and a constant time between follow-up measurements.

### Conclusions

Our results indicate that a longitudinal decrease in eGFR and a longitudinal increase in albuminuria, each independently of each other and of common cardiovascular risk factors, are differentially associated with higher risks of various cardiovascular events and cardiovascular mortality in a large cohort of people with diabetes. These data suggest that early and regular measurement of both markers over time may help to identify people with diabetes at higher cardiovascular risk, providing additional information on specific CVD subtypes.

## Supplementary information

ESM (PDF 335 kb)

## Data Availability

The data used in the present study are available upon request.

## Abbreviations

CKD: Chronic kidney disease
DBP: Diastolic BP
DCS: Hoorn Diabetes Care System
HF: Heart failure
HFpEF: Heart failure with preserved ejection fraction
HFrEF: Heart failure with reduced ejection fraction
MI: Myocardial infarction
SBP: Systolic BP
UACR: Urinary albumin/creatinine ratio
