# Supplementary material for: Kidney function measures and cardiovascular outcomes in people with diabetes: the Hoorn Diabetes Care System cohort
Source: Diabetologia. 2022 Nov 8;66(3):482–94. doi: 10.1007/s00125-022-05826-y (PMC9892144; doi:10.1007/s00125-022-05826-y)
Supplement: Supplementary file 1 — (PDF 335 kb) [file 125_2022_5826_MOESM1_ESM.pdf]

Kidney Function Measures and Cardiovascular Outcomes in People with Diabetes: the Hoorn Diabetes Care System cohort

Elisa Dal Canto[1], Petra Elders[2], Amber A. van der Heijden[3], Adriana J. van Ballegooijen[3], Birgit I. Lissenberg-Witte[3], Femke Rutters[3], Joline W.J. Beulens[3,4].

1 Department of Experimental Cardiology, University Medical Center Utrecht, The Netherlands. 2 Department of General Practice and Elderly Care Medicine, Amsterdam University Medical Center, The Netherlands; 3 Department of Epidemiology and Data Science, Amsterdam University Medical Center, The Netherlands; 3 Julius Center for Health Sciences and Primary Care, University Medical Center Utrecht, the Netherlands.

Electronic Supplemental Material

ESM Table 1: Baseline characteristics of 13,657 individuals with diabetes.

	**Total population**
N	13,657
Age (years)	62.3±12.1
Male sex (%)	53.6
Smoking status	
Current smokers (%)	20.8
Former smokers (%)	37.6
Education level	
Low	42.9
Medium	40.6
High	15.7
Type 2 diabetes (%)	98.9
Type 1 diabetes (%)	0.8
Other diabetes types (MODY, LADA, etc)	0.2
Diabetes duration (years)	0.7 (0.1-3.3)
HbA1c (%)	7.2 ±1.5
**Comorbidities**	
eGFR (ml/min/1.73m^2)	80.4±18.8
UACR (mg/mmol)	0.6 (0.3-1.4)
Arterial Hypertension (%)	54.9
Brachial blood pressure (mmHg)	
Systolic	143±21
Diastolic	81±10
Obesity (%)	44.9
BMI (kg/m^2)	30.2±5.5
Total serum cholesterol (mmol/l)	5.0±1.2
Serum HDL-cholesterol (mmol/l)	1.2±0.3
Serum LDL-cholesterol (mmol/l)	3.0±1.0
**Medications**	
Anti-hypertensive medications (%)	
ACE-ARBs	32.5
Beta-blockers	31.4
Calcium antagonist	14.1
Diuretics	22.4
Lipid-lowering medication (%)	40.0
Anti-hyperglycaemic medications (%)	69.9
Oral anti-hyperglycaemic medications (%)	59.2
Metformin	52.5

Sulfonylureas	23.5
Dipeptidyl peptidase-4 inhibitors	0.5
Glucagon-like peptide 1 receptor agonists	0.1
Sodium-glucose cotransporter 2 inhibitors	0.1
Thiazolidinediones	0.2
Insulin use (%)	10.7

Values are means ± SD, percentages or median (IQR)

ARBs, angiotensin receptor blockers; LADA, latent autoimmune diabetes in adults.

ESM Table 2. eGFR and albuminuria categories of 13,657 individuals with diabetes at baseline.

**eGFR categories [a]**	**%**
G1 (≥ 90 min/ml/1.73m^2)	32.2
G2 (60-90 min/ml/1.73m^2)	54.0
G3a (45-59 min/ml/1.73m^2)	10.5
G3b (30-44 min/ml/1.73m^2)	2.9
G4 (15-29 min/ml/1.73m^2)	0.5
G5 (< 15 min/ml/1.73m^2)	0.1
**Albuminuria categories [a] (based on UACR)**	
A1 (< 3.0 mg/mmol)	85.6
A2 (3.0-30.0 mg/mmol)	12.3
A3 (> 30.0 mg/mmol)	2.2
**Risk categories [a] based on eGFR and Albuminuria**	
Low risk	76.1
Moderately increased	17.8
High risk	4.7
Very high risk	1.4

[a] Kidney function and risk categories are based on the classification proposed by KDIGO 2017 Clinical Practice Guideline for the Evaluation and Management of Chronic Kidney Disease.

ESM Table 3: Baseline characteristics of 13,657 individuals with diabetes stratified by albuminuria categories.

	<3.0 mg/mmol	3.0-30.0 mg/mmol	>30.0 mg/mmol
N	11688	1675	294
Age (years)	61.8±11.9	65.3±12.8	66.7±12.8
Male sex (%)	52.6	58.7	63.8
Smoking status			
Current smokers (%)	19.9	25.9	27.2
Former smokers (%)	38.4	33.4	30.3

Education level			
Low	42.4	46.4	45.7
Medium	41.0	37.3	38.8
High	15.7	15.9	15.4
Type 2 diabetes (%)	98.9	98.8	99.2
Type 1 diabetes (%)	0.8	0.8	0.4
Other diabetes types (MODY, LADA, etc)	0.3	0.4	0.4
Diabetes duration (years)	0.6 (0.1-3.0)	1.2 (0.2-5.7)	2.0 (0.3-7.7)
HbA1c (mmol/mol)	54 ± 8	60 ± 9	62 ± 9
HbA1c (%)	7.1±1.5	7.6 ±1.7	7.8 ±1.7
**Comorbidities**			
eGFR (ml/min/1.73 m^2)	81.3±18.0	76.8±21.7	68.3±24.7
Arterial Hypertension (%)	52.6	67.7	74.5
Brachial blood pressure (mmHg)			
Systolic	141±20	149±23	155±25
Diastolic	80±10	82±11	84±11
Obesity (%)	44.6	46.1	47.8
BMI (kg/m^2)	30.2±5.5	30.3±5.6	30.7±6.1
Total serum cholesterol (mmol/l)	5.0±1.1	5.1±1.2	5.2±1.3
Serum HDL-cholesterol (mmol/l)	1.2±0.3	1.2±0.3	1.2±0.3
Serum LDL-cholesterol (mmol/l)	3.0±1.0	3.0±1.0	3.0±1.1
**Medications**			
Anti-hypertensive medications			
ACE-ARBs (%)	31.2	39.6	52.0
Beta-blocker (%)	30.1	34.2	41.2
Calcium antagonist (%)	12.7	21.0	32.0
Diuretics (%)	22.1	24.7	22.4
Lipid-lowering medication (%)	39.4	43.1	49.3
Anti-hyperglycaemic medications (%)	67.5	78.8	83.0
Oral anti-hyperglycaemic medications (%)	58.3	63.2	60.5
Metformin	51.8	57.2	56.5
Sulfonylureas	22.4	30.0	33.3
Dipeptidyl peptidase-4 inhibitors	0.5	0.7	0.3
Glucagon-like peptide 1 receptor agonists	0.1	0.1	0.0
Sodium-glucose cotransporter 2 inhibitors	0.1	0.0	0.0
Thiazolidinediones	0.3	0.1	0.3
Insulin use (%)	9.2	15.6	22.4

Values represent mean values and standard deviation, percentages or median (interquartile range)

eGFR: estimated glomerular filtration rate. UACR: urinary albumin-to-creatinine ratio. CVD: cardiovascular disease; HF: heart failure; MI: myocardial infarction; HDL: high-density lipoprotein; LDL: low-density lipoprotein; BMI: body mass index; ACE: angiotensin-converting enzyme; ARBs: angiotensin receptor blockers.

ESM Table 4. Hazard ratios of myocardial infarction, heart failure, stroke and coronary heart disease in individuals with diabetes for negative steps of eGFR of 10 ml/min/1.73m^2.

	Model 1			Model 2			Model 3		
	HR	95% CI	P	HR	95% CI	P	HR	95% CI	P
Myocardial Infarction	**1.04**	**1.03,1.18**	**0.004**	**1.11**	**1.03,1.19**	**0.004**	1.07	0.99,1.15	0.075
Heart Failure	**1.14**	**1.01,1.28**	**0.028**	**1.14**	**1.01,1.28**	**0.030**	**1.12**	**1.00,1.26**	**0.050**
Stroke	1.07	0.94,1.22	0.280	1.09	0.95,1.25	0.210	1.10	0.96,1.26	0.170
Coronary Heart Disease	**1.11**	**1.05,1.18**	**<0.001**	**1.11**	**1.05,1.19**	**<0.001**	**1.09**	**1.02,1.16**	**0.014**
Cardiovascular Mortality	**1.24**	**1.14,1.34**	**<0.001**	**1.19**	**1.10,1.29**	**<0.001**	**1.09**	**1.01,1.19**	**0.033**

Model 1 is adjusted for age, sex, SBP, LDL cholesterol, HbA1c, BMI, duration of diabetes, smoking status and education. Model 2 is additionally adjusted for lipid-lowering therapy, use of angiotensin-converting enzyme inhibitor or angiotensin receptor blocker, any treatment for arterial hypertension, use of insulin and use of oral hypoglycemic agents. Model 3 is adjusted as model 2 with the addition of Lg(UACR).

ESM Table 5. Hazard ratios of myocardial infarction, heart failure, stroke and coronary heart disease in individuals with diabetes for Lg(UACR).

	Model 1			Model 2			Model 3		
	HR	95% CI	P	HR	95% CI	P	HR	95% CI	P
Myocardial Infarction	1.12	0.90,1.39	0.290	1.11	0.90,1.39	0.313	1.09	0.88,1.36	0.419
Heart Failure, men	1.04	0.63,1.71	0.885	1.01	0.60,1.68	0.971	0.98	0.60,1.60	0.944
Heart Failure, women	1.41	0.89,2.23	0.138	1.40	0.87,2.24	0.158	1.34	0.84, 2.14	0.220
Stroke	**1.48**	**1.08,2.03**	**0.014**	**1.56**	**1.12,2.17**	**0.009**	1.49	1.06, 2.09	**0.021**
Coronary Heart Disease	1.02	0.831.25	0.860	1.01	0.83,1.24	0.888	0.98	0.80, 1.21	0.903
Cardiovascular Mortality	**2.05**	**1.69,2.48**	**<0.001**	**1.90**	**1.57,2.31**	**<0.001**	**1.80**	**1.48, 2.20**	**<0.001**

Model 1 is adjusted for age, sex, SBP, LDL cholesterol, HbA1c, BMI, duration of diabetes, smoking status and education. Model 2 is additionally adjusted for lipid-lowering therapy, use of angiotensin-converting enzyme inhibitor or angiotensin receptor blocker, any treatment for arterial hypertension, use of insulin and use of oral hypoglycemic agents. Model 3 is adjusted as model 2 with the addition of negative steps of eGFR of 10 ml/min/1.73m^2.

ESM Table 6. Hazard ratios of myocardial infarction, heart failure, stroke, coronary heart disease and cardiovascular mortality in individuals with diabetes stratified by risk categories [a] of eGFR and albuminuria.

	**Myocardial Infarction**			**Heart Failure**			**Stroke**			**Coronary Heart Disease**			**Cardiovascular Mortality**		
N of events		325			127			95			408			686	
Event rate [b] per 1000 person-years		3.08			1.12			0.84			3.72			6.25	
	HR	95% CI	P	HR	95% CI	P	HR	95% CI	P	HR	95%CI	P	HR	95%CI	P
**Model 1**															
Low risk		Reference			Reference			Reference			Reference			Reference	
Moderate risk	1.29	0.97, 1.72	0.088	1.08	0.66,-1.77	0.752	1.16	0.67, 1.99	0.599	1.27	0.97, 1.65	0.079	**1.70**	**1.20, -2.40**	**0.002**
High risk	1.03	0.64, 1.65	0.895	1.78	0.96, 3.31	0.067	1.37	0.68, 2.75	0.383	1.04	0.69, 1.57	0.852	**4.17**	**3.00, -5.80**	**<0.001**
**Model 2**															
Low risk		Reference			Reference			Reference			Reference			Reference	
Moderate risk	1.28	0.93, 1.71	0.105	1.06	0.64, 1.74	0.817	1.18	0.67, 2.07	0.566	1.27	0.97, 1.66	0.077	**1.52**	**1.08, 2.15**	**0.017**
High risk	1.03	0.65,-1.66	0.892	1.72	0.93, 3.19	0.083	1.43	0.69, 2.98	0.335	1.05	0.69, 1.60	0.806	**3.52**	**2.53;, 4.90**	**<0.001**

[a] Risk categories are based on the classification proposed by KDIGO 2017 Clinical Practice Guideline for the Evaluation and Management of Chronic Kidney Disease.

[b] Event rates were calculated as the number of events divided by the total person-years of follow-up, and expressed as the number per 1000 person-years.

Model 1 is adjusted for age, sex, SBP, LDL cholesterol, HbA1c, BMI, duration of diabetes, smoking status and education. Model 2 is additionally adjusted for lipid-lowering therapy, use of angiotensin-converting enzyme inhibitor or angiotensin receptor blocker, any treatment for arterial hypertension, use of insulin and use of oral hypoglycemic agents.

ESM Table 7. Hazard ratios of myocardial infarction, heart failure, stroke, coronary heart disease and cardiovascular mortality in individuals with diabetes stratified by baseline eGFR and albuminuria clinical categories.

	**Myocardial Infarction**			**Heart Failure**			**Stroke**			**Coronary Heart Disease**			**Cardiovascular Mortality**		
N of events		325			127			95			408			686	
	HR	95% CI	P	HR	95% CI	P	HR	95% CI	P	HR	95%CI	P	HR	95%CI	P
**Model 1**															
eGFR > 90 ml/min/1.73m^2		Reference			Reference			Reference			Reference			Reference	
eGFR 60-90 ml/min/1.73m^2	1.21	0.83, 1.52	0.462	1.11	0.63, 1.94	0.718	1.44	0.81, 2.56	0.212	0.93	0.71, 1.23	0.631	0.78	0.53, 1.16	0.226
eGFR < 59 ml/min/1.73m^2	1.37	0.87, 2.17	0.179	**2.16**	**1.04, 4.47**	**0.038**	2.27	0.80, 6.49	0.125	1.12	0.74,1.70	0.595	1.15	0.71, 1.87	0.567
UACR < 3.0 mg/mmol		Reference			Reference			Reference			Reference			Reference	
UACR 3.0-30.0 mg/mmol	0.81	0.58, 1.13	0.213	0.97	0.54, 1.76	0.932	1.10	0.53, 2.29	0.798	0.78	0.57, 1.08	0.134	1.12	0.82, 1.54	0.467
UACR > 30.0 mg/mmol	1.54	0.63, 3.78	0.342	1.02	0.29, 3.60	0.866	1.65	0.22, 10.6	0.628	1.27	0.56, 2.87	0.572	**2.74**	**1.46;5.18**	**0.002**
**Model 2**															
eGFR > 90 ml/min/1.73m^2		Reference			Reference			Reference			Reference			Reference	
eGFR 60-90 ml/min/1.73m^2	1.04	0.77, 1.42	0.779	1.15	0.66, 2.03	0.617	1.40	0.77, 2.39	0.253	0.92	0.70, 1.21	0.539	0.75	0.51, 1.11	0.154
eGFR < 59 ml/min/1.73m^2	1.31	0.82, 2.08	0.256	**2.10**	**1.01, 4.40**	**0.048**	2.13	0.75, 6.59	0.303	1.04	0.68, 1.58	0.850	1.07	0.65, 1.76	0.784
UACR < 3.0 mg/mmol		Reference			Reference			Reference			Reference			Reference	
UACR 3.0-30.0 mg/mmol	0.73	0.52, 1.03	0.072	1.06	0.60, 1.88	0.845	1.08	0.50, 2.19	0.865	**0.69**	**0.50, 0.96**	**0.026**	1.08	0.79, 1.47	0.640
UACR > 30.0 mg/mmol	1.25	0.51, 3.08	0.629	1.10	0.33, 3.71	0.869	1.61	0.24, 12.5	0.760	1.13	1.50, 2.58	0.768	**2.68**	**1.37, 5.26**	**0.004**
**Model 3**															
eGFR > 90 ml/min/1.73m^2		Reference			Reference			Reference			Reference			Reference	
eGFR 60-90 ml/min/1.73m^2	1.00	0.73, 1.37	0.794	1.15	0.65, 2.03	0.637	1.34	0.77, 2.39	0.253	0.87	0.66, 1.15	0.337	0.80	0.54, 1.20	0.277

eGFR < 59 ml/min/1.73m^2	1.26	0.79, 2.02	0.735	**2.13**	**1.02, 4.48**	**0.045**	2.10	0.75, 6.59	0.303	1.01	0.67, 1.54	0.949	1.07	0.65, 1.76	0.787
UACR < 3.0 mg/mmol		**Reference**			Reference			Reference			**Reference**			**Reference**	
UACR 3.0-30.0 mg/mmol	0.73	0.52, 1.03	0.070	0.91	0.50, 1.65	0.755	1.05	0.48;, 2.11	0.890	**0.67**	**0.48, 0.93**	**0.018**	1.05	0.77, 1.44	0.744
UACR > 30.0 mg/mmol	1.17	0.89, 2.84	0.739	0.87	0.24, 3.10	0.866	1.49	0.22, 12.6	0.776	1.12	0.49, 2.54	0.793	**2.26**	**1.12, 4.53**	**0.022**

[a]Event rates were calculated as the number of events divided by the total person-years of follow-up, and expressed as the number per 1000 person-years

Model 1 is adjusted for age, sex, SBP, LDL-cholesterol, HbA_{1c}, BMI, duration of diabetes, smoking status and education. Model 2 is additionally adjusted for lipid-lowering therapy, use of ACE inhibitors or angiotensin receptor blockers, any treatment for arterial hypertension, use of insulin and use of oral hypoglycaemic agents. Model 3 is adjusted as for model 2 with the addition of albuminuria categories for the analysis of eGFR or eGFR categories for the analysis of albuminuria

ESM Table 8. Sensitivity analyses - hazard ratios of myocardial infarction, heart failure, stroke, coronary heart disease and cardiovascular mortality in individuals with diabetes stratified by eGFR and albuminuria clinical categories.

	**Myocardial Infarction**			**Heart Failure**			**Stroke**			**Coronary Heart Disease**			**Cardiovascular Mortality**		
**Baseline analysis**															
N of events		325			127			95			408			686	
Event rate [a] per 1000 person-years		3.08			1.12			0.84			3.72			6.25	
	HR	95% CI	P	HR	95% CI	P	HR	95% CI	P	HR	95%CI	P	HR	95%CI	P
eGFR > 90 ml/min/1.73m^2					Reference									Reference	
eGFR 60-90 ml/min/1.73m^2	**1.52**	**1.10, 2.12**	**0.022**	1.40	0.77, 2.57	0.270	2.53	1.27, 5.03	0.008	**1.67**	**1.23, 2.26**	**0.001**	0.75	0.46, 1.20	0.227
eGFR < 59 ml/min/1.73m^2	**1.69**	**1.09, 2.64**	**0.012**	1.73	0.84, 3.56	0.135	2.34	0.91, 5.94	0.077	**2.01**	**1.34, 3.02**	**<0.001**	1.29	0.76, 2.14	0.349
UACR < 3.0 mg/mmol															
UACR 3.0-30.0 mg/mmol	1.03	0.73, 1.46	0.850	1.26	0.74, 2.12	0.392	1.35	0.77, 2.35	0.293	0.98	0.72, 1.34	0.907	**1.87**	**1.41, 2.47**	**<0.001**
UACR > 30.0 mg/mmol	**1.98**	**1.18, 3.30**	**0.009**	1.81	0.73, 4.55	0.201	1.87	0.72, 4.83	0.195	1.57	0.95, 2.59	0.077	**2.78**	**1.78, 4.34**	**<0.001**
**Excluding CVD**															
N of events		322			112			86			391			603	

Event rate [a] per 1000 person-years		3.08			1.05			0.81			3.77			5.62	
eGFR > 90 ml/min/1.73m^2		Reference			Reference			Reference			Reference			Reference	
eGFR 60-90 ml/min/1.73m^2	**1.55**	**1.11, 2.15**	**0.010**	1.15	0.61, 2.17	0.662	**2.66**	**1.00, 7.07**	**0.004**	**1.69**	**1.24, 2.29**	**0.002**	0.70	0.41, 1.20	0.191
eGFR < 59 ml/min/1.73m^2	**1.63**	**1.03, 2.58**	**0.036**	1.58	0.75, 3.32	0.226	**2.90**	**1.39, 6.03**	**0.044**	**1.93**	**1.28, 2.93**	**<0.001**	1.16	0.65, 2.07	0.610
UACR < 3.0 mg/mmol		Reference			Reference			Reference			Reference			Reference	
UACR 3.0-30.0 mg/mmol	1.05	0.75, 1.48	0.772	1.38	0.80, 2.39	0.248	1.39	0.77 2.49	0.269	0.98	0.71, 1.34	0.885	**1.84**	**1.34, 2.52**	**<0.001**
UACR > 30.0 mg/mmol	**1.91**	**1.12, 3.26**	**0.017**	1.68	0.60, 4.68	0.319	1.69	0.62, 4.58	0.304	1.50	0.90, 2.52	0.120	**3.16**	**1.93, 5.17**	**<0.001**
**Excluding T1D**															
N of events		324			127			94			394			624	
Event rate [a] per 1000 person-years		3.19			1.16			0.86			3.91			5.62	
eGFR > 90 ml/min/1.73m^2					Reference			Reference			Reference			Reference	
eGFR 60-90 ml/min/1.73m^2	**1.53**	**1.10, 2.12**	**0.011**	1.41	0.77, 2.59	0.261	**2.66**	**1.29, 5.48**	**0.008**	**1.67**	**1.23, 2.26**	**<0.001**	0.73	0.45, 1.17	0.188
eGFR < 59 ml/min/1.73m^2	**1.70**	**1.09, 2.66**	**0.020**	1.74	0.85, 3.58	0.129	2.46	0.94, 6.47	0.068	**2.02**	**1.34;3.04**	**<0.001**	1.21	0.72, 2.02	0.467
UACR < 3.0 mg/mmol		Reference			Reference			Reference			Reference			Reference	
UACR 3.0-30.0 mg/mmol	1.04	0.74, 1.46	0.841	1.26	0.74, 2.12	0.392	1.38	0.79, 2.41	0.258	0.99	0.72, 1.35	0.924	**1.85**	**1.40, 2.45**	**<0.001**
UACR > 30.0 mg/mmol	**1.98**	**1.18, 3.31**	**0.010**	1.82	0.72, 4.55	0.202	1.95	0.76, 5.04	0.167	1.57	0.95, 2.59	0.076	**2.83**	**1.80, 4.45**	**<0.001**

[a] Event rates were calculated as the number of events divided by the total person-years of follow-up, and expressed as the number per 1000 person-years.

All analyses are adjusted for age, sex, SBP, LDL cholesterol, HbA1c, BMI, duration of diabetes, smoking status, education, lipid-lowering therapy, use of angiotensin-converting enzyme inhibitor or angiotensin receptor blocker, any treatment for arterial hypertension, use of insulin and use of oral hypoglycemic agents, albuminuria categories for the analysis on eGFR or eGFR categories for the analysis on albuminuria.

Excluding CVD: all patients with any CVD prior to baseline have been excluded. Excluding T1D: patients with type 1 diabetes and other diabetes types other than type 2 have been excluded.

Excluding TIA: from the analysis on cerebrovascular accidents patients who experience transitory ischemic attach have been excluded.

ESM Table 9. Sensitivity analysis - hazard ratios of heart failure in men and women with diabetes patients stratified by albuminuria clinical categories.

	**Men**			**Women**		
	HR	95% CI	P	HR	95% CI	P
**Baseline analysis**						
N of events		69			58	
Event rate [a] per 1000 person-years		1.16			1.06	
UACR < 3.0 mg/mmol		Reference			Reference	
UACR > 3.0 mg/mmol	0.78	0.39, 1.54	0.474	**2.79**	**1.47, 5.28**	**0.002**
**Excluding CVD**						
N of events		57			55	
Event rate [a] per 1000 person-years		1.05			1.04	
UACR < 3.0 mg/mmol		Reference			Reference	
UACR > 3.0 mg/mmol	0.73	0.34, 1.57	0.426	**2.88**	**1.50, 5.53**	**0.001**
**Excluding T1D**						
N of events		69			58	
Event rate [a] per 1000 person-years		1.20			1.10	
UACR < 3.0 mg/mmol		Reference			Reference	
UACR > 3.0 mg/mmol	0.76	0.38, 1.49	0.423	**2.73**	**1.44, 5.19**	**0.002**

[a] Event rates were calculated as the number of events divided by the total person-years of follow-up, and expressed as the number per 1000 person-years.

Because of a very limited amount of HF events within the category of UACR > 30.0 mg/mmol in both sexes, for the stratified analysis categories of UACR=3.0-30.0 and UACR > 30.0 have been combined into one category.

Effect modification by sex for the association between heart failure and albuminuria categories: interaction dummy for UACR category >3.0 mg/mmol: P=0.010.

All analyses are adjusted for age, sex, SBP, LDL cholesterol, HbA1c, BMI, duration of diabetes, smoking status, education, lipid-lowering therapy, use of angiotensin-converting enzyme inhibitor or angiotensin receptor blocker, any treatment for arterial hypertension, use of insulin, use of oral hypoglycemic agents and eGFR categories.

ESM Table 10. Complete-case analysis**.** Hazard ratios of myocardial infarction, heart failure, stroke, coronary heart disease and cardiovascular mortality in individuals with diabetes stratified by eGFR and albuminuria clinical categories.

	**Myocardial Infarction**			**Heart Failure**			**Stroke**			**Coronary Heart Disease**			**Cardiovascular Mortality**		
N of events		325			127			95			408			686	
Event rate [a] per 1000 person-years		3.08			1.12			0.84			3.72			6.25	
	HR	95% CI	P	HR	95% CI	P	HR	95% CI	P	HR	95%CI	P	HR	95%CI	P
**Model 1**															
eGFR > 90 ml/min/1.73m^2		Reference			Reference			Reference			Reference			Reference	
eGFR 60-90 ml/min/1.73m^2	**1.59**	**1.14, 2.21**	**0.006**	1.36	0.74, 2.51	0.316	**2.47**	**1.25, 4.99**	**0.009**	**1.71**	**1.26, 2.32**	**<0.001**	0.81	0.50, 1.29	0.374
eGFR < 59 ml/min/1.73m^2	**1.82**	**1.18, 2.82**	**0.007**	1.72	0.83, 3.53	0.139	2.34	0.95, 5.73	0.063	**2.08**	**1.40, 3.10**	**<0.001**	1.64	0.98, 2.73	0.058
UACR < 3.0 mg/mmol		Reference			Reference			Reference			Reference			Reference	
UACR 3.0-30.0 mg/mmol	1.09	0.80, 1.49	0.591	1.16	0.69, 1.94	0.583	1.29	0.76, 2.19	0.346	1.05	0.76, 1.42	0.802	**1.93**	**1.44, 2.58**	**<0.001**
UACR > 30.0 mg/mmol	**1.71**	**1.01, 2.91**	**0.046**	1.73	0.70, 4.24	0.234	1.47	0.58, 3.77	0.419	1.66	0.99, 2.79	0.055	**3.72**	**2.37, 5.83**	**<0.001**
**Model 2**															
eGFR > 90 ml/min/1.73m^2		Reference			Reference			Reference			Reference			Reference	
eGFR 60-90 ml/min/1.73m^2	**1.58**	**1.14, 2.20**	**0.008**	1.38	0.75, 2.52	0.300	**2.58**	**1.30, 5.13**	**0.007**	**1.70**	**1.26, 2.31**	**<0.001**	0.74	0.46, 1.18	0.204
eGFR < 59 ml/min/1.73m^2	**1.84**	**1.18, 2.86**	**0.006**	1.70	0.83, 3.47	0.145	**2.53**	**1.01;, 6.37**	**0.048**	**2.08**	**1.39, 3.10**	**<0.001**	1.34	0.81, 2.22	0.251
UACR < 3.0 mg/mmol		Reference			Reference			Reference			Reference			Reference	
UACR 3.0-30.0 mg/mmol	1.06	0.75, 1.49	0.851	1.13	0.64, 1.99	0.661	1.38	0.80, 2.41	0.257	1.02	0.75, 1.40	0.891	**1.76**	**1.32, 2.37**	**<0.001**
UACR > 30.0 mg/mmol	**1.98**	**1.15, 3.40**	**0.013**	1.66	0.60, 4.63	0.331	1.77	0.68, 4.59	0.241	1.62	0.96, 2.71	0.072	**3.28**	**2.10, 5.11**	**<0.001**
**Model 3**															
eGFR > 90 ml/min/1.73m^2		Reference			Reference			Reference			Reference			Reference	
eGFR 60-90 ml/min/1.73m^2	**1.57**	**1.13, 2.18**	**0.014**	1.37	0.75, 2.51	0.301	**2.56**	**1.28, 5.10**	**0.007**	**1.69**	**1.25, 2.29**	**0.001**	0.72	0.45, 1.16	0.175
eGFR < 59 ml/min/1.73m^2	**1.75**	**1.12, 2.73**	**0.008**	1.65	0.81, 3.36	0.163	2.39	0.93, 6.15	0.070	**2.01**	**1.34, 3.02**	**<0.001**	1.20	0.72, 2.00	0.473
UACR < 3.0 mg/mmol		Reference			Reference			Reference			Reference			Reference	

UACR 3.0-30.0 mg/mmol	1.05	0.75, 1.49	0.772	1.13	0.64;, 1.98	0.670	1.39	0.79, 2.41	0.249	1.01	0.74, 1.39	0.944	**1.70**	**1.27, 2.29**	**<0.001**
UACR > 30.0 mg/mmol	**1.87**	**1.08, 3.23**	**0.023**	1.57	0.56, 4.38	0.390	1.68	0.62, 4.54	0.306	1.49	0.88, 2.53	0.136	**2.92**	**1.84, 4.63**	**<0.001**

[a]Event rates were calculated as the number of events divided by the total person-years of follow-up, and expressed as the number per 1000 person-years

Model 1 is adjusted for age, sex, SBP, LDL-cholesterol, HbA_{1c}, BMI, duration of diabetes, smoking status and education. Model 2 is additionally adjusted for lipid-lowering therapy, use of ACE inhibitors or angiotensin receptor blockers, any treatment for arterial hypertension, use of insulin and use of oral hypoglycaemic agents. Model 3 is adjusted as for model 2 with the addition of albuminuria categories for the analysis of eGFR or eGFR categories for the analysis of albuminuria

ESM Table 11. Complete case-analysis**.** Hazard ratios of heart failure in men and women with diabetes stratified by albuminuria clinical categories.

	Men			Women		
N of events		69			58	
Event rate [a] per 1000 person-years		1.16			1.06	
	HR	95% CI	P	HR	95% CI	P
**Model 1**						
UACR < 3.0 mg/mmol		Reference			Reference	
UACR > 3.0 mg/mmol	0.48	0.20, 1.26	0.136	**2.80**	**1.41, 5.49**	**0.003**
**Model 2**						
UACR < 3.0 mg/mmol		Reference			Reference	
UACR > 3.0 mg/mmol	0.47	0.18, 1.21	0.118	**2.74**	**1.38, 5.43**	**0.004**
**Model 2**						
UACR < 3.0 mg/mmol		Reference			Reference	
UACR > 3.0 mg/mmol	0.47	0.18, 1.22	0.273	**2.79**	**1.42, 5.47**	**0.003**

[a] Event rates were calculated as the number of events divided by the total person-years of follow-up, and expressed as the number per 1000 person-years.

Because of a very limited amount of HF events within the category of UACR > 30.0 mg/mmol in both sexes, for the stratified analysis categories of UACR=3.0-30.0 and UACR > 30.0 have been combined into one category.

Effect modification by sex for the association between heart failure and albuminuria categories: interaction dummy for UACR category > 3.0 mg/mmol: P=0.010.

Model 1 is adjusted for age, sex, SBP, LDL cholesterol, HbA1c, BMI, duration of diabetes, smoking status and education. Model 2 is additionally adjusted for lipid-lowering therapy, use of angiotensin-converting enzyme inhibitor or angiotensin receptor blocker, any treatment for arterial hypertension, use of insulin and use of oral hypoglycemic agents. Model 3 is adjusted as model 2 with the addition of eGFR categories.

eGFR: estimated glomerular filtration rate. UACR: urinary albumin-to-creatinine ratio

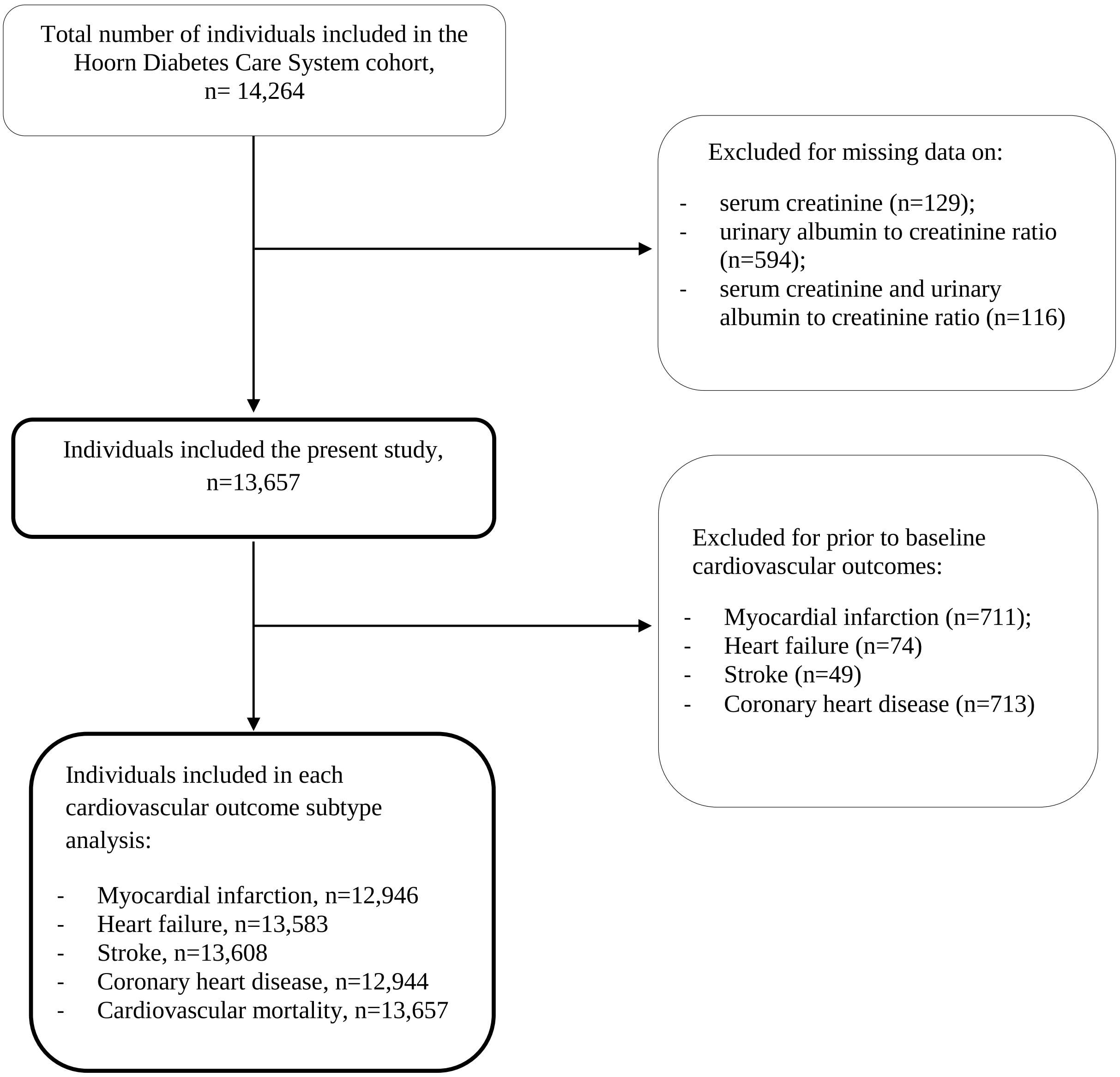

Supplemental Figure 1. Flow chart of the study population selection
